# How do adolescents with depression experience improvement in psychodynamic psychotherapy? A qualitative study

**DOI:** 10.1186/s12888-019-2080-0

**Published:** 2019-03-21

**Authors:** André Løvgren, Jan Ivar Røssberg, Liv Nilsen, Eivind Engebretsen, Randi Ulberg

**Affiliations:** 1 Division of Mental Health and Addiction, Oslo University Hospital, P.O. box 4959, N-0424 Nydalen, Oslo Norway; 2 University of Oslo, Institute of Clinical Medicine, P.O. box 1171, 0318 Blindern, Oslo Norway; 3 University of Oslo, Institute of Health and Society, P.O. box 1130, 0318 Blindern, Oslo Norway; 4 Vestfold Hospital Trust, Division of Mental Health, Research Unit, P.O. box 2169, 3125 Tønsberg, Norway

**Keywords:** Adolescent depression, Psychodynamic psychotherapy, Experiences of improvement, Therapeutic change, Qualitative study

## Abstract

**Background:**

There is emerging evidence for the effectiveness of psychodynamic psychotherapy for depressive disorders. However, we know less of how this relation-focused therapy mode is experienced and what the patients themselves identify as helpful. Hence, the purpose of this study is to explore adolescents’ experiences of factors promoting improvement in psychodynamic therapy.

**Methods:**

Eight female patients participating in a Norwegian study on psychodynamic therapy, the First Experimental Study of Transference Work – In Teenagers (FEST-IT), were included. The participants were offered a total number of 28 sessions. Semi-structured qualitative interviews about experiences with therapy were then conducted and analysed with systematic text condensation and hermeneutic interpretation.

**Results:**

The analysis revealed four main themes. ‘Exploring oneself’ comprises autonomy and acknowledgment, openness, insight and acceptance of oneself. ‘Therapist relation and characteristics’ includes confidence and trust in and support from the therapist as well as having a trustworthy and experienced therapist. ‘Focusing on everyday life’ includes learning and practical orientation. ‘Time factors’ refers to duration and frequency.

**Conclusions:**

Together with a supportive and listening therapist, the adolescents improve by exploring themselves within the frames of a time-limited treatment period. Improvement seems to be experienced through better relations to oneself and to others and by finding one’s place in the family, or at school. Adolescents value problem solving and help with concrete challenges. Hence, therapy should be tailored to the needs of adolescents with depression and incorporate the challenges they face in their everyday life.

**Trial registration:**

ClinicalTrials.gov. Id: NCT01531101. Date of registry: 8 February 2012, retrospectively registered.

## Background

Major depressive disorder (MDD) is a prevalent psychiatric disorder associated with significant disability, mortality and economic burden [1]. Depression is strongly associated with reduced quality of life and capability to work or study and impedes relations in family and social life. In the US, the 12-month prevalence of major depressive episodes (MDE) equates to 11.3% [2]. In Norway, Sund et al. [3] found the prevalence of life-time depression to be 23% among adolescents aged 12–15. Adolescent depression enhances the risk of later depression and impaired psychosocial functioning in adult life [4]. This calls for high priority in identifying and treating depression in adolescents.

Psychotherapy for adolescent depression often relies on psychodynamic therapy (PDT) or cognitive-behavioural therapy (CBT). Both treatments are shown to be effective and helpful [5–7]. However, less is known of how therapy works and what the helpful factors are. To achieve a better understanding of how therapy contributes to improvement and what improvement means to those receiving help, the experiences of the patients are central. To explore this field further, qualitative methods are suitable and requested [8–12].

Although there are several case studies and descriptions of patients in PDT, systematic qualitative studies of how patients experience this intervention are few. Nilsson et al. [13] compared experiences of change in PDT and CBT in *adult patients*. Satisfied PDT patients reported a feeling of understanding oneself better as the most distinctive experience of change. Changes came around when talking about and reflecting upon oneself and then piecing things together again. Gostas et al. [14] compared patients’ experiences in PDT and CBT with a focus on the contract and the process within the two approaches. Analysing the empirical data, they found the methods to be quite similar. The results of the study underscore building up trust, adapting techniques to the patients’ needs and capacities and contract negotiations as important. Patients’ knowledge of their own needs, difficulties and desire for change have to influence and affect the contract and the process of psychotherapy.

Some studies have qualitatively explored *adolescents’ and young people*’*s* needs in therapy or counselling. Gibson et al. [15] grouped the findings from 12 qualitative studies into three areas, as follows: First, adolescents want a genuine sense of connection to their therapist. This relationship is valued as highly important and is preferred to contain a degree of mutuality. Young people want their therapist to be non-judgmental, empathic and caring. Secondly, they want to be able to freely express their thoughts and emotions. Finally, young people seem particularly conscious of their lack of power in relation to adults. Their need for autonomy and control within the therapeutic encounter, and issues such as privacy and confidentiality, are important to the adolescents. This requires an equal relationship over which the adolescents are able to exercise control [15].

Henriksen [16] interviewed adolescents with various diagnoses after different types of successful outpatient treatment. Helpful factors included talking about difficult emotions, strange thoughts or crucial events. Scrutinising their destructive thoughts, preconceptions and actions was important as well as the therapist challenging such thoughts and actions. Also viewed as important was adjusting the therapy to the adolescents’ preferences.

Some of these 13 studies have included adolescents with depression in their sample, but none of the studies are restricted to PDT. This paper contributes to the evidence base as, to the best of our knowledge, no studies have qualitatively examined how adolescents with a depressive disorder experience improvement through PDT. To lessen this knowledge gap is of major importance. MDD is frequent among adolescents and seems to increasing worldwide, and PDT is a well-known and widespread treatment for depression. Nevertheless, depression seems to be undertreated [17], and dropout rates seem high [18–20]. Accordingly, establishing the best possible treatment for adolescents with a depressive disorder is of major importance, and increasing knowledge on how therapy works is crucial. Consequently, the main focus of the present study is to explore how adolescents with depression experience improvement through PDT.

## Method

### Design

Participants were recruited from the Norwegian randomised controlled trial (RCT) study the ‘First Experimental Study of Transference Work – In Teenagers (FEST–IT)’ [21]. The present study is qualitative and nested in this RCT. The FEST–IT study aimed to explore the effects of relational interventions in psychodynamic psychotherapy for adolescents with depression. The study recruited patients, aged 16–18 years, among adolescents referred to private practice or to public child and adolescent outpatient departments in the southeast of Norway. This area covers both urban and rural places. The inclusion criteria was a diagnosis of MDD as defined in the Diagnostic and Statistical Manual of Mental Disorders, 4th Edition (DSM-IV). The exclusion criteria were generalised learning difficulties, pervasive developmental disorder, psychosis or substance abuse. The FEST–IT study offered 28 sessions of therapy and used the published manual [22] of the ‘Improving Mood with Psychoanalytic and Cognitive Therapies (IMPACT)’ study [23]. The manual focuses on techniques aimed at helping young people overcome developmental and relational problems as well as emphasising the role of the interpretation of unconscious conflicts, insight and the concepts of internal working models. With the agreement of the adolescent, parallel work with parents was included. Antidepressant medication could be added in severe cases according to Norwegian national guidelines. The patients were randomised to two treatment groups, those with and those without transference interventions. In the transference group the therapists encouraged exploration of the patient-therapist relationship. In both groups, general psychodynamic techniques were used. Follow-up was conducted at post-treatment and one year after treatment termination.

### Data collection

An interview guide was developed for the in depth interviews. The main questions covered the adolescents’ experiences of therapy in general, what within the therapy sessions was helpful, or not so helpful, and how therapy affected important relations and areas in the adolescents’ everyday lives. Thirteen adolescents were invited to participate in the present qualitative interview study when they met for their evaluation. There was no purposive sampling, and they were recruited as the inclusion to the RCT was still going on. Consequently, the qualitative interviewers did not know whether the interviewed patients had received therapy with or without transference interventions. Furthermore, the interviewers were blind to the outcome assessments at evaluation and did not know whether the patients had improved or deteriorated during the interventions. The interviewers did not know from which of the therapists the patients had received treatment. The qualitative interviewers conducted nine individual in-depth interviews. One patient had completed only four sessions of her therapy, had no experience of improvement and is not included in the present report. We have no reason to believe that the nine interviewed adolescents are non-representative of the study population in general. The first author conducted the interviews either alone or together with the third author, both experienced psychiatric nurses. In line with the Declaration of Helsinki [24], attention was paid to whether, in any way, the interview situation should be difficult or straining. The audiotaped interviews lasted from 40 to 60 min. The first author transcribed verbatim the interviews immediately after they took place. All transcriptions were anonymised.

### Participants

All of the interviewed participants were female ethnic Norwegians attending secondary school, and their mean age at inclusion to FEST–IT was 17 years. All met the criteria for MDD, and the baseline level of depression had a mean of 29.7 (range 22–37), whereas for the study population as a whole it was 28.7 (range 10–58, SD 9.0), as measured with the Beck Depression Inventory II (BDI-II) [25]. One patient had been diagnosed with a personality disorder (Axis II, SIDP-interview for DSM-IV). Seven of the interviewed patients met with a male therapist, and two met with a female therapist. Registered attendance varied from 19 to 28 sessions (mean 26, 8 sessions). Housing varied evenly among living with one or both parents or alternating between them.

### Analysis

We see the interviewer as a co-producer of the data who, together with the participant, affects the interview process. This interactive approach means that the data are seen not only as objective information coming from inside the patient but are also produced in a unique encounter between the persons involved, who act and react upon each other [26]. To help organise and categorise the transcribed interviews, the computerised program NVivo 11 was used (QSR International Pty Ltd.). To narrow the focus, the first author read the transcribed interviews guided by the question: ‘What does the adolescent say about how they have experienced improvement?’ This reduced the totality of the transcribed material. The texts were read and re-read several times and were coded into categories that best represented the meaning stated, as seen in relation to the interview as a whole. This also meant that the same part of the text could be used to illustrate different themes and represent various categories. This depended on the context of the utterance as well as the interests and pre-understanding of the authors. The reading of the transcripts and the analysis and categorising processes were discussed among the authors and revisions made. This developed over time, making the interpretations less vulnerable to individual preferences and biases. In this hermeneutic circle of interpretation, we identified differences and similarities among the adolescents’ experiences. We tried to avoid reducing the complexity and the variations in the material. This led to a close reading of the nuanced descriptions of individual processes of improvement experienced by the adolescents.

The development of themes and categories followed the procedure of systematic text condensation (STC) [27]. STC involves analytic reduction with specified shifts between the de-contextualisation and re-contextualisation of data. We first searched for a general impression of the transcripts as a whole, with our awareness tuned to the voice of the participants. Then, we first identified preliminary themes and secondly identified and sorted out meaning units. Meaning units are de-contextualised text fragments from all the interviews containing information about the research question. These were coded and labelled and grouped into the themes identified in step one. We then extracted meanings from all the codes and condensed them into descriptions. Finally, an authentic quotation was given to exemplify and illustrate certain aspects of the meanings embedded in the codes [27]. In the present study, all eight participants are represented, each with at least two quotations. To preserve anonymity, the quotations are not attributed to specific participants.

## Results

The adolescents experienced several factors promoting improvement in therapy. We organised them into four themes, each with several subthemes. ‘Exploring oneself’ describes an ongoing process during the therapy sessions and includes insight into and acceptance of oneself, autonomy and acknowledgement as well as openness. ‘Therapist relation and characteristics’ describes confidence and trust in and support from the therapist as well as having a therapist who is trustworthy and experienced. ‘Focusing on everyday life’ refers to learning and practical orientation as important for combining therapy with everyday life. ‘Time factors’ comprises both the frequency of sessions and the duration of therapy (see Table 1).

**Table 1:** Factors promoting improvement: themes and main categories

Themes	Main categories
Exploring oneself	Autonomy and acknowledgment Openness Insight and acceptance of oneself
Therapist relation and characteristics	Confidence, trust and support Trustworthy and experienced
Focusing on everyday life	Learning Practical orientation
Time factors	Duration and frequency

### Exploring oneself

Exploring oneself means how the adolescents expand their understanding of themselves. This appears when they integrate new perspectives into their feelings, thoughts and behaviour. They see themselves from another perspective and get alternatives to the way they normally see themselves.

#### Autonomy and acknowledgment

The concept of autonomy mainly refers to independence from others’ judging or opinions of oneself and to different aspects of the management of control. One adolescent said: *‘The therapist was very good at helping me with my image of myself’.* This was made possible simply through the therapist’s behaviour: *‘He just sat there and listened to me’.* This helped her a lot more than friends that used to actively argue against her negative opinions about herself. Through the therapists listening, she became more independent of how her friends viewed her and her situation. Autonomy also appeared as important for the adolescents towards the therapist. Central here was to exercise control over what to say and when to say it and not being forced to talk about a subject until the adolescents felt comfortable in doing so. In addition, therapy contributed to autonomy in everyday life: *‘In one way, it helped me to take actions, speak up, do this-do that, and stop paying attention to things’.* However, autonomy and control over when to say what was also experienced in a more demanding way. This appeared when the therapist did not wait patiently for the adolescent to open up about a subject, and thus the adolescent experienced a form of pressure: *‘It was something that I tried to keep deep inside of me. And then, I was not allowed to do that. And that was very relieving, in a way being forced to really feel it’.*

Acknowledgment refers to valuing and affirming the adolescents and their situation. The therapists, the adolescents and the therapy itself all contributed to this acknowledgement. Entering therapy was in itself experienced as a crucial acknowledgment of oneself as a suffering person. This came about as the adolescent was valued as suffering heavily enough to require help and their difficulties not being devalued as a typical teenage-thing. Receiving a diagnosis could also be an acknowledgement as it offers an explanation to the way one is, as if saying: ‘*It’s okay; there is a reason for the way you are’.* This eased the burden from expectations telling the adolescent to pull herself together.

An opposite way of acknowledging occurred when the therapy debunked the adolescents’ fear of being out of their mind or crazy. Acknowledgment through normalising the situation and problems was also imparted by, or more precisely, attributed to, the therapist: *‘I got confirmation that I had good reasons for being sad and down… he somehow confirmed that “You have a tough and difficult time, but here we can talk about it”’.* An active and acknowledging therapist, in contrast to a careful listening therapist, was also crucial for improvement: *‘To me, it was very rewarding coming to a therapy session and saying: “Hi, I did it!” I subsisted a lot on the acknowledgement I would then receive. It’s very fun when somebody gives you praise for fulfilling something difficult’.*

The adolescents’ own acknowledgements which contributed to improvement in therapy varied in content and direction. First, it was very helpful to acknowledge that they themselves had problems and actually were struggling. The importance of not denying or belittling their difficulties and painful feelings but rather acknowledging them as real was in a distinct way stated by a patient explaining what really helped her in therapy: *‘Actually, one of the most important things I learned was “Okay, I’m not doing well” because I wouldn’t quite admit it to myself’.*

In addition, the adolescents acknowledging not only their problems or neglected feelings but also their dreams and wishes also facilitated improvement. To take these seriously was for one experienced as a turning point in therapy. Taking something seriously meant for her to verbalise and tell the therapist that she actually knew what she wanted. By putting it into words, hearing it loud and admitting what she really wants to do with her life, made the wishes in a way real and true: *‘To say out loud that this is what I want in one way puts reality and goals into it’.*

#### Openness

To be open, or opening up, refers among other things to the adolescents’ basic experience of talking with another person. They describe how therapy opened up their feelings and thoughts, seeing this as very important. Sometimes the therapist took the active part: *‘He made me realise things, and just opened me up’.* Often the adolescents described themselves as taking the active part as they opened themselves up. The therapist asked questions that motivated this process. Especially helpful were questions about what they felt in different situations. Going deeper into what they felt simultaneously expanded their repertoire and the nuances of their feelings. Being asked questions no one else had asked before also made the adolescent more open to themselves. This, in turn, could make them more open towards others. In different ways, openness was described as an obvious necessary, but demanding process, and several also described challenges in being open with the therapist. Another adolescent put it this way when talking about what really helped her in therapy: *‘Just to open up. There’s nothing scary about opening up, and people need someone to talk to’.*

#### Insight and acceptance of oneself

The interviewees described insight as recognising one’s needs and as becoming more aware of their feelings. Thinking through and analysing their feelings when they appeared made this possible. When going deeper into their feelings they made several discoveries about themselves, and as one put it: *‘The key was that I learned to think more, go deeper into things. Then first, I realised,* *“Wow; this is actually the way I am”’.*

Several of the adolescents experienced this intimate and complex connection between thinking and feeling as ways to insight. Some emphasised working with their feelings as more important than focusing on their thinking. Others experienced the opposite, achieving insight when they became more aware of their thinking. For one, the work with ‘the feeling and the thinking’ was experienced as gaining control: *‘My way of thinking changed. My feelings are just my feelings. But due to my way of thinking, I got better control over my feelings’.*

In addition to such internal endeavours, there was also the experience that insight into oneself led to better understanding of their relation to other people: *‘I didn’t understand how people could be the way they were, when I was not at all like them’.* This connecting of one’s own psychological and emotional state to an appropriate understanding of the behaviour of other people was seen as an important achievement in therapy: *‘I understood why the world was going around’.*

Acceptance of oneself describes how increased knowledge into, and differentiation between, their feelings and needs helped the adolescents to a more sensitive form of caring for themselves. And not to ‘*have too high expectations of oneself*’, as one said. This became clear in their relations to family members, friends or other pupils at school: *‘During therapy I became better at recognising what’s best for me. And then to take that space, and actually do what’s good for me’.* For one adolescent, struggling to stop harming herself, it became important to recognise that she could identify herself with something other than the harm she did to herself. Frightened of being empty inside or being of no importance, therapy helped her to put some of *herself* into the core of who she was: *‘And that made me realise I exist whether I cut myself or not’.*

Taking other peoples’ perspective into account and considering oneself with the same tolerance with which one look upon others also facilitated acceptance of oneself. One adolescent, who was afraid of making mistakes or looking dumb in front of her classmates, found it very helpful to understand that nobody cared about whether or not she made some mistakes: *‘Nobody cares about it, except me’.* This made her realise that she was too critical of herself. Another way acceptance of self was put into words was allowing oneself or to give oneself permission to suffer and have problems and show it to others. Showing the therapist that there were problems in their life made it easier to show it and talk about it with others. One patient, often feeling that she did not get anything out of therapy, nevertheless experienced ‘the allowing of self’ as one of the most important achievements from therapy: *‘Even though I’ve often felt that I didn’t achieve anything in that therapy, I was always told, “You are allowed to be who you are”. That is very cool to keep in mind. I think about that every day’.*

### Therapist relation and characteristics

The relation to the therapist plays a crucial role in therapy. Experiences of confidence, trust and support are central in this helpful relation. Additionally, the therapist should be trustworthy and experienced.

#### Confidence, trust and support

The adolescents pay a great deal of attention to the therapist. They wonder who the person in front of them really is; they notice differences in the therapist’s way of being, changes in, for example, hairstyle, the way he or she talks and so on. They try to know their therapist. It is a one-way relationship, and as one said: *‘It is a person who is close to me, but for whom I am just one out of many. And that, also, is very special’.* In this relationship, the patients nevertheless experienced safety and comfort facilitated by the fact that the adolescent did not have to fear the consequences of what they said: *‘All the time, in a way I tested the limits of what I could say without being afraid of being judged’.* Trust and confidence were crucial for a safe therapeutic relationship, expressed by one this way: *‘In one way, I can say whatever I want, without what I say backfiring on me’.*

Being vulnerable and crying in front of another person can be relieving. Feeling irrational and destroyed, the importance of trust and confidence was clearly described by one adolescent: *‘Having someone else helps a lot. It’s a place where it is okay to be weak, it’s okay not to know what you are talking about, what you should do, who you are or anything’.* Another way confidence and trust were experienced was more directly connected to what the therapist was saying. One adolescent who really seemed to rely on the therapist said: *‘I took all that he said to heart. I had such strong faith in him that I felt everything he said was right. Because he was one that I trusted’.* Another adolescent experienced a reduction in feelings of suicidality and anxiety as well as a strong decrease of panic attacks shortly after entering therapy. Her explanation for this improvement centred on the safety of therapy, not necessarily on the therapist: *‘I felt that being in therapy, no matter whatever happens in there, also can give safety, or help a little, in itself’.*

Another way in which the relational work contributed to improvement was through support. Although, as one of the adolescents saw it, you basically have to do the work yourself, the relation played an important role: *‘What I needed was the guidance and support from a person with a little more competence than me’.* Support from the therapist was for another adolescent the only real support she experienced in her process of deciding whether to quit school and move in another direction in her life. Feeling all alone, without support from family or friends in what was most important to her, therapy became the only place where she experienced support: *‘It was very satisfactory feeling that somebody felt it was a good choice for me, that I should do it, and understands it and actually supports me fully and entirely’.* This support made things less lonely and easier to stand up for when her choice was questioned.

Central in the relation, and important for improvement and progress in therapy, were the roles into which the adolescents placed their therapist. One adolescent looked upon her therapist as an ordinary person. Being a good girl, she tried to be nice and polite in shaping the relationship with the therapist. This caused her to avoid talking about what she saw as difficult or problematic. She did not want the therapist to think in a negative way about herself or people she cared about. Gradually realising that this pleasing attitude was hindering her in speaking freely, she progressed when she changed her view of the therapist: *‘I actually began to look upon him as a therapist and not just an ordinary human being I was to make a good impression upon. This was very important for the whole treatment’.*

Another adolescent made an opposite change in her relation to the therapist. Her experience was of a silent therapist, just sitting and listening to her. The focus on her and her problems sometimes made her feel she was getting worse. A turning point arose when she realised that the therapist was not just a typical therapist but quite a nice man with a life of his own. She then gradually acknowledged the therapist as an ordinary human being she could trust: *‘I then, actually, saw him more as a friend I could confide in’.*

While the former patient needed the ordinary person to become a therapist, the latter needed the therapist to become an ordinary person. Changing their views on the role of the therapist was crucial as it helped the adolescents to develop themselves through the further relation with their therapist.

#### Trustworthy and experienced

When describing how the therapist contributes to improvement, the adolescents emphasised a therapist that in different ways is dependable, experienced and trustworthy: *‘He seemed so experienced and talked with such self-confidence. So I became quite convinced, like “okay, what he says is actually true”’.* The same patient experienced a crucial turning point in therapy when the therapist opened himself up a little, giving a focus other than on her and her problems: *‘He disclosed some of his own experiences. And I felt that was the real turning point between the two of us. And then I actually saw him more as a friend I could confide in’.* Another adolescent attributed what made her trust and rely on the therapist more to the role and position: *‘It is something with him being educated… it’s his job, in a way. It has a lot to do with the authority he had as a therapist, I think’.* One adolescent felt that the therapists’ gender had great influence. She felt much better off with her male therapist than her former female therapist. She explained this as being partly because she had expected a female therapist and partly because the one she got was good at talking: *‘He was somehow good at articulating and expressing himself and could somehow ask the right questions’.* He was experienced as a reassuring therapist in that he was quiet until she began to talk. The adolescents need for being in focus and to have another person’s time and attention was important. This was for one of the adolescent’s the main experience of what the therapist had done and, actually, what she felt she needed in therapy: *‘It was a person sitting there listening to me. And just listened to me and only wanted to help. One that actually sat there listening and talking’.*

### Focusing on everyday life

The adolescents regarded improvements in life outside of therapy as important as well. Learning and practical orientation seem to be crucial factors when integrating therapy into everyday life.

#### Learning

The adolescents experienced learning in the close relationship with their therapist. They used the relation to practice on how to take oneself seriously or how to take care of oneself in an appropriate way. They tried to apply experiences and learnings from therapy in their everyday life: *‘Something that is often helpful for me, which I also learned there, is to talk afterwards, when one has calmed dawn’.*

They experienced learning about themselves and distinguishing oneself from others as well as about difficult feelings such as anger. Considering how to prevent relapse and learning about feelings, thoughts and situations where symptoms of depression occur was recognised by one adolescent as very useful: *‘If I in a way now get a kind of negative thought, I know what to do with it’.* Another adolescent put it this way when explaining how she managed to stop behaviour that was not in her interest: *‘It was not me that wanted to do it. It was “the sick me”. I became very good at distinguishing the sick part of me from the healthy part. And that I am still very happy for’.*

#### Practical orientation

Helpful experiences which the adolescents all seemed to share was when therapy became practical and focused on concrete challenges in their everyday life. This could also include becoming conscious of the way they think and then getting help for, and actually making, concrete changes in their way of thinking. One adolescent who had lost contact with friends and family members due to her rejection of them became convinced by the therapist that the friends wanted to be with her because they actually loved her, not because they felt pity for her: *‘He made me understand that the negative thoughts were wrong. And that made me make contact with them again. So all those I’d lost contact with I now have back again’.* Another patient found that her way of thinking changed by doing a form of therapy homework. Not thinking of problems before going to bed but rather thinking of nice things was something she continued to do after ending therapy: *‘I still use those things very much in my everyday life. Concrete suggestions I got in therapy’.* Another adolescent with periods of having great difficulty getting out of bed, getting dressed and facing the day experienced improvement in therapy this way: *‘It was when talking about things and getting some suggestions on concrete practical things one could do to get started. And that was what I felt as the most important to me’.* Also crucial for improvement was receiving explanations for their situation and understanding the reasons behind and causes of why they got angry or sad or why they were depressed, achieved through detailed questions such as what in particular had made them feel, or react, exactly the way they did in this or that concrete situation. One adolescent explained what she felt therapy had helped her with in the following way: *‘It was to handle problems I had in my everyday life and in a way figure out where the core problem of everything was. Find out why I become depressed and why I was so sad all the time’.* Another illuminated how therapy, as a place where one could get answers, created positive expectations. When having a difficult period in between the therapy sessions, she could think: *‘I’m looking forward to going back and talking about this. Why it is the way it is’.* Another described explanations and answers she had received in therapy in terms of tools: *‘I felt I got some good tools. I kind of learned or understood why I think the way I do. It opened up a lot the way I think and made me understand more of why I felt the way I felt in different situations’.*

### Time factors

Time factors refers to two dimensions of helpful experiences, the frequency of the sessions and the duration of the therapy period. For different reasons, the adolescents expressed satisfaction with the duration of the therapy, which was initially 28 weeks. One adolescent who needed some breaks between sessions and stretch out her therapy over a one-year time span experienced relapse during therapy: *‘I think it was fine that it lasted for such a long period because suddenly things were going well. But then I got worse again’.* Tailoring therapy to the adolescents’ situation was also discussed by another patient. She was allowed to continue her useful treatment after the original 28 weeks due to difficult changes in her life: *‘If it had stopped where it was supposed to, I think I would have become very empty inside and very, very alone with the situation I was in at that moment’.* Another said*: ‘Getting the opportunity for a long enough treatment period was very useful for me, because I don’t think I would have achieved much from a shorter therapy period’.* Going to a predictable and continuous therapy once a week was essential for her improvements in therapy: *‘I somehow always had that thing. I think that was just as useful as the therapy itself’.* The helpful aspects of therapy as fixed, weekly appointments was expressed by another this way: *‘To keep it going every week, having a fixed schedule has helped quite a lot. It has been a very good way to make the everyday life less demanding’.*

## Discussion

**Fig. 1:**
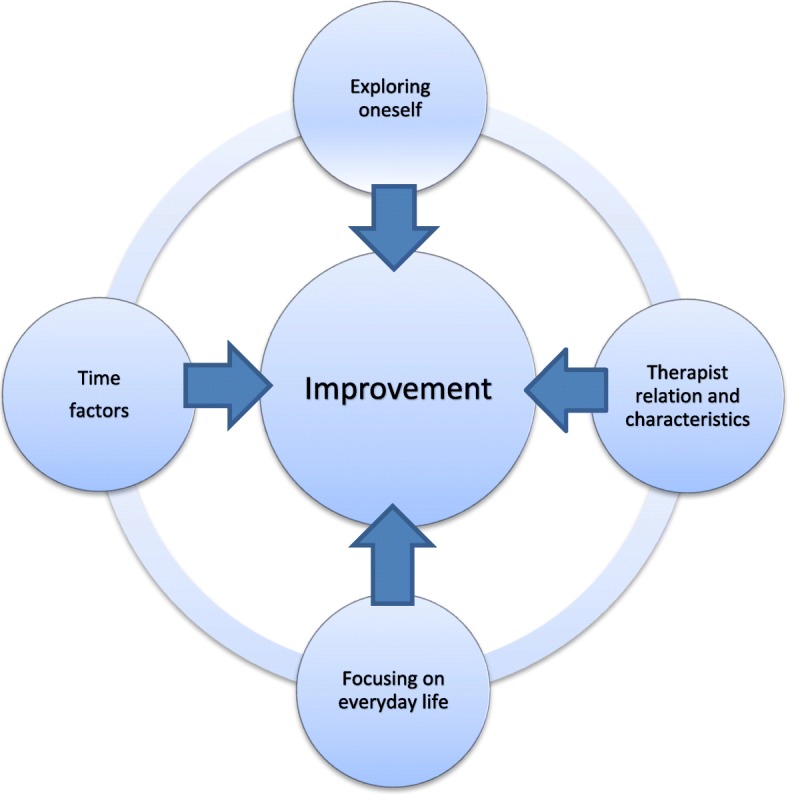
The figure reveals the different aspects of experienced improvement in PDT for adolescents

The present study revealed four themes important for the adolescents’ improvement, each with several subthemes: ‘Exploring oneself’ describes an ongoing process during the therapy sessions and includes insight into and acceptance of oneself, autonomy and acknowledgement as well as openness. ‘Therapist relation and characteristics’ describes having confidence and trust in and support from the therapist as well as having a therapist who is trustworthy and experienced. ‘Focusing on everyday life’ refers to learning and practical orientation as important for combining therapy with everyday life. ‘Time factors’ comprises both the frequency of sessions and the duration of therapy. Our findings are in line with the focus in the treatment manual [22]. The patients experienced improvement through increased insight (Exploring oneself) and the relation to the therapist (Therapist relation and characteristics). The experience from therapy intervened with everyday life (Focusing on everyday life). Over time (Time factors), the patients developed more adequate internal working models, and experienced improvement. Therapists should pay attention to these aspect in therapy for adolescents with depression. The four themes are made up of dynamic elements and are visualised in Fig. 1. We conceptualise this as ‘dynamics of improvement’ and will discuss some of the core elements below.

### Exploring oneself

‘Exploring oneself’ covers insight, acceptance of self, autonomy, acknowledgement and openness. Paying attention to the adolescent’s experience and situation, in contrast to being more structured and task-focused in the initial phase of therapy, is found to facilitate greater participation later on in therapy [28]. In the present study, exploring oneself contributed to improvement through an expanded understanding of the self. It also seems that the adolescents’ new understanding not only makes them more aware of themselves and who they are but that it has the potential to promote concrete improvements in their everyday life as well. The intrapersonal exploring of oneself in combination with its effects at the interpersonal level is of major importance. Central to the concept of autonomy in the present study is to become more independent of the experience of others’ judging of oneself and the managing of control. The managing of control mainly refers to when to say what in therapy and not being forced to talk about issues the patient is not ready for. This is in some contrast to the findings by Gibson et al. [15], where exercising of control is exemplified by an adolescent who ended counselling when she did not like what her counsellor was saying. The concepts of autonomy and control are central in both these studies. The meanings and actions attributed to these concepts, however, are quite different. This is of importance when understanding the adolescents’ experiences.

Control and autonomy seem to be central factors facilitating adolescent therapy, as demonstrated in Binder et al. [29] and underscored in Carver et al. [30] and Sauter et al. [31]. Interestingly, in our material autonomy and the exercising of control are experienced in combination with both ‘being forced to’ and ‘not being allowed to’ avoid difficult themes or feelings. The exercising of autonomy contributes to improvement when the adolescents choose to give in to the therapists’ power and influence. Therapy is established as a place where power and pressure are exercised. In Gibson and Cartwright’s [32] discussion of the possible pitfalls of client power and agency (defined as the capacity of the young client to construct themselves as active in counselling within social constraints), power seems to be considered as something that is used to exercise control in therapy and where the therapist’s power risks disempowering the adolescent. Our findings add important nuances to this. The power of the therapist is experienced and described as therapeutic and contributes to improvement. The productive or therapeutic power is contextual, not something the adolescent, or the therapist, brings into therapy and uses in a superior or independent way. Hence, the patient’s situation, the therapist and their relation as well as the society’s view of adolescence [32] should be considered when trying to understand what power and the exercising of control are an expression of.

### Therapist relation and characteristics

The relation to the therapist is important and, with youth, the therapeutic relationship is found to have moderate to large effects on treatment outcome [33]. In the present study, ‘therapist relation and characteristics’ refers to having confidence and trust in and support from the therapist as well as having a therapist who is trustworthy and experienced. These factors of the therapeutic relationship are of major importance for improvement. The importance of a supportive and non-judging therapist is in line with previous studies [29, 34], and the supportive nature of the therapist is also found to be important for adolescents in their willingness to collaborate in therapeutic interventions [35].

In the present study, the adolescents varied in regard to the degree to which they noticed and were aware of their therapist. When an adolescent strongly needed to be in focus and have the therapist’s time and attention, the presence of the therapist was hardly noticed at all. However, the adolescents were in need of different therapeutic characteristics, and a comparable example exists in the findings of Gibson et al., where the adolescents in counselling wanted a relationship more of a friendship than a professional relationship [15]. The dichotomisation of the therapeutic relationship into opposites such as friendship and a professional relationship is in the present study experienced as more complex and paradoxical. For one patient, the relationship first became helpful and therapeutic, and as we see it professional, when the therapist used the self-disclosing strategy. However, the adolescent experienced this turning point as gaining a friend to confide in. Hence, having a professional relationship may be experienced as it’s contrary, namely, as having a friendship. This must be taken into consideration for therapists working with adolescents with depression.

A society’s view of adolescence will affect the expectations the adolescents encounter. In the study by Binder et al. [29] of how adolescents prefer their therapists to interact with them, the authors assume adolescence as a period where, among others, a sense of being unique and special is central. This might cause conflicts, and a central role for the therapist is to provide a space different from the closeness in a child-parent relationship. Central in this ‘conflict-discourse’ of adolescence is striving for autonomy and uniqueness and to become an individual independent from others [29]. The present study does not necessarily contradict this but rather points in another direction. The adolescents improve when therapy helps them to find their place within their families and social surroundings. They seem not particularly occupied with themselves as adolescents or as having a genuine need for being unique or special. The present study shows more of a picture of adolescents who want to be included and considered as grownups with responsibility and opinions that are valued. The present study shows the participants’ need for support and help in finding their individuality and uniqueness as steps towards a broader goal: better relations to important others and themselves. They want to live an ordinary life without conflicting relations or troublesome symptoms. A focus on these factors in therapy contributed to improvement. As the adolescents put into words how therapy contributed to improvement, they view themselves in relation to their social surroundings. When they give meaning to what improvement means, they place themselves in a discourse of responsible and conscientious individuals. The adolescents negotiate expectations from family or social surroundings in relation to their own needs and desires. They do this with care for themselves and for those they are to become more independent of and unique to.

The therapist is central in this process, and, in the present study, helpful therapist characteristics relate to the role and position of the therapist and to the therapist as a person. Self-doubt as a therapist combined with a high degree of self-affiliation as a person is, admittedly in adults, found to be particularly helpful [36]. We believe this to be the case with young persons as well. The adolescents’ need for a person they can confide in, depend upon and have the attention of requires a therapist who believes oneself to be such a person.

### Focusing on everyday life

Learning and practical orientation were emphasised by the adolescents as important for their improvement. These occurred when therapy became directed towards concrete challenges in their everyday life. Dunne, Thompson and Leitch [37] found that adolescents place less importance on factors such as cognitive tasks and problem solving and rather emphasise emotional exploration as helpful. Their study reports on questionnaire and interview data from counselling in an all-boys school in Ireland. Hence, all 11 participants were male adolescents, and the therapist in the study was trained in client-centred therapy and reality therapy and was also the study’s chief researcher [37]. Such differences in gender, design and context, compared to the present study, may partly explain the different results. Nevertheless, these two studies question an assumption of female adolescents as emotionally oriented and male adolescents as practically oriented. However, in a qualitative meta-analysis the importance of using strategies and guidance are underscored. Reporting on helpful factors in school-based counselling, both relational factors such as talking openly and being listened to and problem-solving activities and strategies for dealing with problems were reported as helpful [38]. Hence, therapy should regard both problem solving and emotional exploration as ways to improvement and weight the balance between them in cooperation with the adolescents.

The value of guidance and problem solving is underscored in Hanley and Noble’s overview of how children and youth view therapy [39]. The practical orientation the adolescents in the present study reported as crucial to improvement included getting explanations for their situations and answers to what was happening to them. Why they no longer felt happy or were so sad. To change their thinking and to get advice on what to do or say to better get along with oneself and others also helped the adolescents to improve and increased their social wellbeing and functioning. To some extent, this practical, down-to-earth oriented improvement seems for the adolescents to have a specific goal: to feel normal and to have a sense of uncomplicated social belonging with good relations at school, in their families and with friends. Such effects, beyond symptom relief, seem to be supported by Pattison and Harris’s investigation into the effectiveness of counselling children and young people [40].

The adolescents in the present study improved by talking about emotions and thoughts and by this getting to know themselves better. By exploring their thoughts and actions, it became clearer what alternative ways to handle their problems could be. Henriksen (2013) found it helpful when the therapist challenged the adolescents’ strange thoughts or actions [16]. In our material it seems like the therapists’ challenges were experienced in a more indirect way. Suggestions from the therapist of what to do or say in everyday life, or questions about their thinking, seemed to reinforce the challenges the adolescents put to themselves in their efforts to achieve improvement. Therapists need to be aware that they might put forth challenges without intending to. More research on the role of advice and suggestions, and how to best deliver these to create improvement, is also underscored in Hayes and Brunst [41], and the present study adds nuances to this issue.

### Time factors

Time as a helping factor relates to the experience of receiving a sufficient number of sessions, which for the patients in the present study comprised 28. Fixed weekly encounters with the therapist, and periods with infrequent appointments, helped in regard to improvement. The factors of frequency of sessions and duration of the therapy period contributed to improvement when they were adapted to the shifting needs in the adolescents’ everyday life. This is in correspondence with the findings by Gibson et al. [15]. As time passes within and between therapy sessions, it seems the adolescents experienced having a possibility from which they, with crucial help from the therapist, are allowed to mature and grow. This almost makes time itself into an independent factor that contributes to improvement. A firmly established therapy period contributes to providing the necessary stability in a confusing and difficult period in the adolescent’s life.

### Limitations and strengths

Contextual factors are essential to understand why and how particular experiences and meanings from therapy come into being. In the discussion, we compare experiences generated from different contexts and circumstances. The experiences are summed up into categories, making comparisons with other studies possible. However, this risks weakening the voice of the patients by neglecting unique experiences that do not easily fit into those categories [42] and by leaving outside the situated meaning attributed to the experiences. The present study included only female participants, and, due to its qualitative design, the findings are not generalizable in a statistical sense for the population at large. Our findings are helpful for therapists in the tailoring of therapy. Further studies, however, are needed.

## Conclusion

Eight female adolescents’ experiences of improvement in PDT are explored. Together with a supportive and listening therapist, they achieved improvement by exploring themselves within the frames of a fixed treatment period. Improvement seems to have been experienced by better relations to oneself and to others and by finding one’s place in the family, or at school. They highly valued help with concrete challenges and problem solving. We see this as crucial for therapists to be aware of in therapy. The tailoring of therapy to the needs of adolescents with depression should carefully consider the individual challenges they face in their everyday life.

## Abbreviations

CBT: Cognitive behavioural therapy
DSM-IV: Diagnostic and Statistical Manual of Mental Disorders - IV
FEST–IT: First Experimental Study of Transference Work – In Teenagers
MDD: Major depression disorder
MDE: Major depressive episodes
PDT: Psychodynamic therapy
RCT: Randomised controlled trial
REC: Regional Committees for Medical and Health Research Ethics in Norway
SIDP: Structured Interview for DSM-IV Personality
STC: Systematic text condensation
